# Growth Differentiation Factor-8 (GDF8)/Myostatin Is a Predictor of Troponin I Peak and a Marker of Clinical Severity after Acute Myocardial Infarction

**DOI:** 10.3390/jcm9010116

**Published:** 2019-12-31

**Authors:** Alexandre Meloux, Luc Rochette, Maud Maza, Florence Bichat, Laura Tribouillard, Yves Cottin, Marianne Zeller, Catherine Vergely

**Affiliations:** 1 Laboratoire Physiopathologie et Epidémiologie Cérébro-Cardiovasculaires (PEC2, EA 7460), Université de Bourgogne-Franche-Comté, UFR des Sciences de Santé; 7 Bd Jeanne d’Arc, 21000 Dijon, France; alexandre.meloux@u-bourgogne.fr (A.M.); luc.rochette@u-bourgogne.fr (L.R.); maud.maza@chu-dijon.fr (M.M.); florence.bichat@chu-dijon.fr (F.B.); yves.cottin@chu-dijon.fr (Y.C.); marianne.zeller@u-bourgogne.fr (M.Z.); 2 Department of Cardiology, University Hospital of Dijon, 21000 Dijon, France; laura.tribouillard@chu-dijon.fr

**Keywords:** GDF8, myostatin, AMI, troponin

## Abstract

Objective: Growth differentiation factor-8 (GDF8), also known as myostatin, is a member of the transforming growth factor-β superfamily that inhibits skeletal muscle growth. We aimed to investigate the association between GDF8 and peak troponin I levels after acute myocardial infarction (AMI). Methods: All consecutive patients admitted from June 2016 to February 2018 for type 1 AMI in the Coronary Care Unit of University Hospital of Dijon Bourgogne (France) were included in our prospective study. Blood samples were harvested on admission, and serum levels of GDF8 were measured using a commercially available enzyme-linked immunosorbent assay kit. Results: Among the 296 patients with type 1 AMI, median age was 68 years and 27% were women. GDF8 levels (median (IQR) = 2375 ng/L) were negatively correlated with age, sex and diabetes (*p* < 0.001 for all). GDF8 levels were higher in patients with in-hospital ventricular tachycardia or fibrillation (VT/VF) than those without in-hospital VT/VF. GDF8 was positively correlated with troponin I peak (r = 0.247; *p* < 0.001). In multivariate linear regression analysis, log GDF8 (OR: 21.59; 95% CI 34.08–119.05; *p* < 0.001) was an independent predictor of troponin I peak. Conclusions: These results suggest that GDF8 levels could reflect the extent of myocardial damage during AMI, similar to peak troponin I, which is currently used to estimate infarct size. Further studies are needed to elucidate the underlying mechanisms linking the GDF8 cytokine with troponin I levels.

## 1. Introduction

Patients with acute myocardial infarction (AMI) have a high rate of mortality, and the risk of fatal events is highest in the first hours following onset. The severity of AMI, which is usually determined early on with the measurement of circulating troponins, has a major impact on the development of late AMI consequences such as heart failure. Therefore, precise and rapid assessment of the severity of AMI critically affects treatment choices and patient prognoses. Recently, there has been interest in the potential role of new biomarkers for the assessment of severity in the early stages of AMI, with a particular focus on NT-pro-natriuretic peptide (NT-proBNP), heart-type fatty acid binding protein (hFABP) and circulating cytokines such as growth differentiation factor-15 [1].

Growth differentiation factor-8 (GDF8), also known as myostatin, is a member of the transforming growth factor-β (TGF-β) superfamily. GDF8 shares many structural similarities with other members such as growth differentiation factor-11 [2,3]. GDF8 is mainly expressed in skeletal muscles, particularly during the development period but also in adulthood, and is considered a negative regulator of muscle growth [4]. Genetic inhibition of myostatin leads to an increase in skeletal muscle mass and triggers a hyper-muscular phenotype in mammals [5,6]. In the heart muscle, GDF8 is expressed in fetal and adult myocardium [7], and its expression is increased in cardiac diseases such as advanced heart failure [8] or congenital heart disease [9]. Following experimental myocardial infarction, GDF8 is up-regulated in cardiomyocytes surrounding the infarcted area [7] and its concentration rapidly increases in the circulation [10]. However, the role of GDF8 during the acute phase of AMI in humans is poorly understood.

The aim of our study was to evaluate GDF8/myostatin levels in patients admitted for AMI, and to investigate the associations between GDF8 and markers of AMI severity such as troponin.

## 2. Methods

### 2.1. Patients

The methods and design of the French Regional Observatoire des Infarctus de Côte-d’Or (RICO) survey have been previously described [11]. From June 2016 to February 2018, all consecutive patients admitted to the coronary care unit of the Dijon University Hospital (France) for type 1 AMI were prospectively included. Type 1 MI is defined as an acute atherothrombotic coronary event resulting in the formation of an intra-luminal thrombus (plaque rupture, ulceration, erosion or coronary dissection) [12]. The present study is in agreement with the ethical guidelines of the Declaration of Helsinki. All of the participants provided consent prior to inclusion, and the Ethics Committee of the University Hospital of Dijon approved the protocol (BIOCARDIS-2016–9205AAO034S02117).

### 2.2. Data Collection

Patient characteristics were obtained at hospital admission. These included cardiovascular risk factors and history, and clinical and biological data. Risk scores were calculated (GRACE score and SYNTAX score). Blood samples were collected on admission to measure serum C-reactive protein (CRP), creatinine, creatine kinase peak, troponin Ic peak, NT-proBNP, blood lipids, glucose and hemoglobin. eGFR was calculated using Chronic Kidney Disease-EPIdemiology Collaboration formula (CKD-EPI). Echocardiographic data such as left ventricular ejection fraction (LVEF) were recorded. Finally, in-hospital events were documented, including death, cardiovascular death, re-infarction, stroke, development of heart failure and ventricular tachycardia or fibrillation (VT/VF).

### 2.3. Determination of Serum GDF8

Blood samples were collected on admission from a vein in the arm, centrifuged at 4 °C to isolate the serum, and samples were stored at −80 °C until use. Median (IQR) time from symptom onset to blood sampling was 16(8–30) hours. Serum GDF8 was measured in duplicate using a commercially available Quantitine kit (DGDF80, R&D systems, MN). The minimum detectable concentration was 2.25 ng/L, and the coefficient of variation between duplicates did not exceed 10%.

### 2.4. Statistical Analysis

Dichotomous variables are expressed as *n* (%) and continuous variables as mean ± SD or median (interquartile range). A Kolmogorov–Smirnov test was performed to test the normality of continuous variables. For non-normally distributed variables (i.e., NT-proBNP), they were log transformed. The Mann-Whitney test or Student’s *t* test was used to compare continuous data, and the Chi 2 test or Fisher’s test was used for dichotomous data, as appropriate.

Pearson correlation analyses (for normally distributed variables) or Spearman correlation analyses (one or two non-Gaussian variables) were performed. The threshold for significance was set at 5%.

Bivariate linear regression analyses were used to adjust GDF8 with age.

Multivariate logistic regression models were built to estimate in-hospital VT/VF and troponin Ic peak based on significant variables in univariate analysis. The inclusion threshold was set at 5%.

SPSS version 12.0.1 (IBM Inc, Armonk, NY, USA) was used for all of the statistical tests.

## 3. Results

The baseline characteristics of the study population are shown in Table 1. Predictors of GDF8 are shown in Table 2. GDF8 levels were significantly associated with age, sex and diabetes. Clinical data showed an association with systolic and diastolic blood pressure, STEMI, heart failure and GRACE risk score. Moreover, GDF8 was strongly correlated with CRP, creatine kinase peak, troponin Ic peak, NT-proBNP and LDL-cholesterol, as well as creatinine clearance and acute statin medication.

### 3.1. Baseline Characteristics

Among the 296 included patients, eighty-one (27%) were female. The median age was 68 years, 178 (60%) had hypertension, 117 (40%) had hypercholesterolemia, 75 (25%) had diabetes and 85 (29%) are active smokers. Median GDF8 was 2375 (1640–3347) ng/L.

### 3.2. Associations between GDF8 Levels and in-Hospital Development of Ventricular Tachycardia or Fibrillation

**Figure 1:**
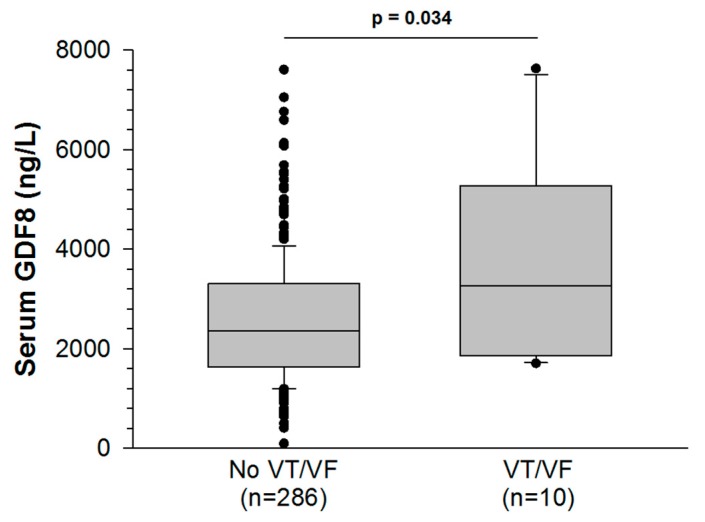
Serum growth differentiation factor-8 (GDF8) levels rise more in AMI patients with ventricular tachycardia or fibrillation (VT/VF) than AMI patients without VT/VF.

Ten patients (3%) developed VT/VF during their hospital stay. GDF8 levels were higher in these patients than in those who did not experience VT/VF (2565 ± 75 vs. 3852 ± 642 ng/L, *p* = 0.034, Figure 1). To assess VT/VF risk, a GDF8 cut-off value of 2878 ng/L was established with a receiver operating characteristic (ROC) curve analysis. The value was rounded to 2800 ng/L to improve clinical relevance. The area under the curve (AUC) was 0.697 (*p* = 0.034) and the sensitivity and specificity were good (70% and 66%, respectively). Among patients with GDF8 > 2800 ng/L (112/296), the risk of developing in-hospital VT/VF was higher than in patients with GDF8 < 2800 ng/L (184/296) (*p* = 0.046). The other relevant biomarkers (CK and peak troponin Ic) showed similar associations with the outcome (VT/VF): the respective AUC were 0.717 (*p* = 0.027) and 0.698 (*p* = 0.034), and the cut-off values were 400.5 UI/L and 7.6 ng/mL. Sensitivity and specificity were respectively 100% and 44% for CK and 100% and 43% for peak troponin IC. Both CK and troponin Ic were significantly associated with VT/VF in logistic regression analysis (CK peak: OR (95% CI): 6.034 (1.684–21.621) and troponin Ic peak: OR (95% CI): 2.751 (1.079–7.019)).

### 3.3. Associations between GDF8 Levels and Peak Troponin Ic

GDF8 was correlated with peak troponin Ic (r = 0.247; *p* <0.001). Patients with high (i.e., supramedian) GDF8 levels had a trend toward an increased risk of TV/FV compared with patients who had lower (i.e., inframedian) GDF8 levels (4.8% vs. 2%). Moreover, the troponin peak was much higher (X3) in patients with a supramedian GDF8 level, as shown in Table 3. In univariate analysis, diabetes (OR 11.82, 95% CI −3.49–43.03; *p* = 0.095), smoking (OR 11.34, 95% CI 0.78–45.40; *p* = 0.043), left ventricular ejection fraction <40% (OR 19.53, 95% CI 10.88–87.76; *p* = 0.012), GRACE risk score (OR 0.16, 95% CI 0.16–0.76; *p* = 0.003), and time to admission (OR 0.01, 95% CI −0.02–0.001; *p* = 0.065) and log GDF8 (OR 21.59, 95% CI 34.08–119.05; *p* <0.001) were associated with the prediction of troponin Ic peak. In multivariable analysis, log GDF8 remained associated to the prediction of troponin Ic peak, after adjustment for confounding factors (Table 4).

## 4. Discussion

In our study, GDF8 levels were shown to be negatively associated with older age, and positively with female sex; these results corroborate existing clinical data. For instance, previous studies have shown that GDF8 levels were highest in men in their 20s and statistically declined throughout subsequent decades [13]. Indeed, in men, serum GDF8 increases slightly with age until 57 years and then decreases [14]. In both the “Heart and Soul” and the HUNT3 cohorts, GDF11/8 levels were lower in older participants [15]. In patients aged 60 years and older, a recent study has shown that women had higher GDF8 plasma levels than men and that the circulating plasma GDF8 was negatively associated with muscle function [16].

In the present work, we also observed correlations between GDF8 levels and traditional cardiovascular risk factors such as diabetes, increased systolic and diastolic blood pressure, increased LDL cholesterol and CRP. The role of GDF8 in regulating tissue glucose uptake has been documented both in experimental [17] and clinical studies [18,19]. Blocked GDF8 expression in mice resulted in increased insulin signaling and better insulin sensitivity in skeletal muscle [20]. Therefore, in patients with insulin resistance, GDF8 inactivation is a potential target for the prevention of risk factors associated with the development of ischemic cardiovascular diseases. The clinical data are sparse for hypertension, cholesterol levels and CRP, but one experimental study has demonstrated that GDF8 deletion in a mouse model of metabolic syndrome resulted in increased muscle mass and prevented an increase in blood pressure [21]. Inactivation of GDF8 in in *Ldlr-/-* mice was shown to protect against the development of insulin resistance, proatherogenic dyslipidemia and aortic atherogenesis [22].

The main findings of the present study involve the association of GDF8 with the markers of AMI severity such as ST-elevation myocardial infarction (STEMI), occurrence of complicating heart failure, GRACE risk score, CK peak, NT-proBNP, and troponin levels. In multivariable analysis, log GDF8 was associated with the prediction of troponin I peak, even after adjustment for age. Moreover, among patients with the highest GDF8 levels (>2800 ng/L), the risk of developing in-hospital VT/VF was higher. To our knowledge, this is the first time that GDF8 has been associated with clinical severity in the acute phase of MI. Previous studies in sheep found that GDF8 was expressed in the fetal and adult heart and was localized in the cardiomyocytes and Purkinje fibers [7]. Furthermore, after experimental myocardial infarction, GDF8 expression was upregulated in the cardiomyocytes surrounding the infarcted zone. Studies performed in mice have shown that GDF8 was upregulated in the heart as early as 10 min after coronary artery ligation, reaching peak expression in tissue between 24 h and 1 month following the acute event. In the serum of the mice, GDF8 levels also promptly and steadily increased [10]. Indeed, elevated circulating levels of GDF8 have been observed in several types of serious myocardial diseases such as anthracycline-induced cardiotoxicity [23] and in experimental [24,25,26] and clinical heart failure [8,9,27]. In particular, serum GDF8 levels were shown to have predictive value for the severity of chronic heart failure and to be a predictor of adverse prognosis in these patients [27]. In myocardial infarction, both the destruction of the cardiac tissue and the up-regulation of its expression may account for the elevated levels found in serum. Consequently, it has been suggested that the heart could function as an endocrine organ promoting skeletal or myocardial muscle wasting, inducing cardiac muscle weakness [25]. In fact, the absence of GDF8 in GDF8-deficient mice subjected to myocardial infarction seemed to protect the heart, possibly by limiting the extent of fibrosis and improving survival [28]. We suggest here that during the course of AMI, GDF8 is produced and released by the cardiac tissue proportionally to the severity of the ischemia. GDF levels may therefore be strongly associated with peak troponin, but also with the occurrence of complications such as heart failure or ventricular arrhythmias. Of course, the estimation of myocardial damage is complex and might not be only reflected by one circulating factor such as GDF8 and/or troponin peak. Hemodynamic measurements, expansion index, and other exams as such as magnetic resonance imaging are necessary to quantify the extent of the infarct, the myocardial tissue loss and fibrosis after AMI [29,30]. Further studies should be conducted to evaluate whether GDF8 could be a predictor of poor outcomes after AMI, in particular those related to skeletal or myocardial muscle wasting.

## 5. Study Limitations

The first limitation of our study is the small number of patients who developed VT/VF (*n* = 10), limiting statistical power. The second limitation is the monocentric nature of the study with a subsequent selection bias. However, the strong association between GDF8 and the prediction of troponin I peak was supported by results of univariate regression analysis (*p* < 0.001) and the enduring significance after adjustment for determinants (*p* < 0.001). In future, these preliminary results need to be confirmed in larger studies.

## 6. Conclusions

To conclude, our original results suggest that GDF8 levels could reflect the extent of myocardial damage during AMI, similar to peak troponin I, which is currently used to estimate infarct size. Further studies are needed to elucidate the underlying mechanisms linking the GDF8 cytokine with troponin I levels.

## Figures and Tables

**Table 1:** Baseline characteristics.

	N (%) or Median (IQR) N = 296
**Risk factors**	
Age, y	68 (58–78)
Female	81 (27%)
BMI, kg/m²	26 (24–30), *n* = 295
Hypertension	178 (60%)
Diabetes	75 (25%)
Hypercholesterolemia	117 (40%)
Family history of CAD	74 (25%)
Current smoking	85 (29%)
**Cardiovascular history**	
CAD	53 (18%)
Stroke	17 (6%)
Chronic kidney disease	16 (5%)
**Clinical data**	
LVEF, %	55 (50–60), *n* = 294
LVEF <40%	22 (7%)
HR, bpm	76 (64–87), *n* = 283
SBP, mmHg	142 (123–165), *n* = 275
DBP, mmHg	82 (70–94), *n* = 274
STEMI	143 (48%)
HF	56 (19%)
GRACE risk score	141 (116–170), *n* = 268
ICU length of stay, *d*	3 (3–4), *n* = 290
Coronary angiography	294 (99%)
SYNTAX score	12 (7–18), *n* = 284
Multivessel disease	184 (63%)
Percutaneous coronary intervention	252 (86%)
**Biological data**	
GDF8 relative expression	2375.0 (1640.0–3346.7)
CRP > 3 mg/L	158 (54%)
Creatinine, µmol/L	79 (68–95), *n* = 295
eGFR CKD, mL/min	82.7 (65.9–95.5), *n* = 295
eGFR CKD < 45 mL/min	34 (12%)
CK peak, UI/L	583 (195–1483), *n* = 291
Troponin Ic peak, ng/mL	15.00 (3.21–70.00), *n* = 295
Nt-ProBNP, pg/mL	394 (93–1588), *n* = 295
LDL cholesterol, g/L	1.24 (0.92–1.53), *n* = 293
HDL cholesterol, g/L	0.50 (0.40–0.60), *n* = 293
Total cholesterol, g/L	2.06 (1.70–2.35), *n* = 293
Triglycerides, g/L	1.21 (0.84–1.76), *n* = 293
Glycemia, mmol/L	6.80 (5.80–8.63), *n* = 295
**In-hospital events**	
Death	7 (2%)
Cardiovascular death	6 (2%)
Recurrent MI	7 (2%)
Stroke	2 (1%)
HF	75 (26%)
VT or VF	10 (3%)
**Chronic medications**	
Antiplatelet therapy	24 (8%)
Aspirin	72 (24%)
ARB	63 (21%)
ACE inhibitor	58 (20%)
Statin	92 (31%)
Beta blocker	83 (28%)
Diuretic	57 (19%)
**Acute medications**	
Antiplatelet therapy	283 (96%)
Aspirin	290 (98%)
ARB	36 (12%)
ACE inhibitor	179 (60%)
Statin	270 (91%)
Beta blocker	207 (70%)
Diuretic	65 (22%)

Data are expressed as *n* (%) or median (25th and 75th percentiles). *n*: number; GDF8: growth differentiation factor 8; BMI: body mass index; CAD: coronary artery disease; LVEF: left ventricular ejection fraction; HR: heart rate; HF: Heart failure; SBP: systolic blood pressure; DBP: diastolic blood pressure; STEMI: ST segment elevation myocardial infarction; GRACE: Global Registry of Acute Coronary Events; ICU: Intensive Care Unit; CRP: C-reactive protein; CK: creatine kinase; NT-proBNP: N-terminal pro-brain natriuretic peptide; LDL: low density lipoprotein; HDL: high density lipoprotein; ARB: angiotensin receptor blockers; ACE: angiotensin converting enzyme.

**Table 2:** Association between GDF8 levels and study variables (*n* = 296).

		Patients (n = 296)	GDF8 Relative Expression or r	*p* Value
CV risk factors				
Age (years)		68 (58–78)	−0.26	<0.001
Female	Yes	81 (27)	2002 (1284–2785)	<0.001
	No	215 (73)	2554.9 (1759–3489)	
BMI (kg/m²)		26 (24–30)	0.08	0.191
Hypertension	Yes	178 (60)	2247 (1532–3321)	0.063
	No	118 (40)	2585 (1756–3381)	
Diabetes	Yes	75 (25)	1946 (1429–2621)	<0.001
	No	221 (75)	2574 (1751–3526)	
Hypercholesterolemia	Yes	117 (40)	2501 (1697–3397)	0.396
	No	179 (60)	2311 (1621–3304)	
Current smoking	Yes	85 (29)	2482 (1689–3396)	0.306
	No	211 (71)	2256 (1633–3320)	
Cardiovascular history				
CAD	Yes	53 (18)	2209 (1477–2991)	0.189
	No	243 (82)	2426 (1688–3381)	
Stroke	Yes	17 (6)	1742 (1070–2636)	0.062
	No	279 (94)	2431 (1679–3372)	
Chronic kidney disease	Yes	16 (5)	2712 (1315–3416)	0.885
	No	280 (95)	2368 (1663–3346)	
Clinical data				
LVEF		55 (50–60)	−0.05	0.396
HR (bpm)		76 (64–87)	−0.06	0.304
SBP (mmHg)		142 (123–165)	0.14	0.022
DBP (mmHg)		82 (70–94)	0.22	<0.001
STEMI	Yes	143 (48)	2748 (1802–3445)	0.001
	No	153 (52)	2141 (1519–2973)	
Heart failure	Yes	56 (19)	2018 (1252–2775)	0.006
	No	238 (81)	2511 (1695–3413)	
GRACE risk score		141 (116–170)	−0.22	<0.001
ICU stay length (days)		3 (3–4)	−0.01	0.877
Biological data				
CRP ≥ 3 mg/L	Yes	158 (54)	2102 (1471–3197)	<0.001
	No	136 (46)	2722 (1957–3525)	
Creatinine clearance (CKD EPI) (mL/min)		83 (66–96)	0.15	0.010
CK peak (UI/L)		583 (195–1493)	0.26	<0.001
Peak troponin Ic (ng/mL)		15 (3–70)	0.25	<0.001
NT-proBNP (pg/mL)		394 (93–1588)	−0.27	<0.001
Glucose (mmol/L)		7 (6–9)	−0.02	0.684
LDL cholesterol (g/L)		1.2 (0.9–1.5)	0.25	<0.001
HDL cholesterol (g/L)		0.5 (0.4–0.6)	0.07	0.211
Triglycerides (g/L)		1.2 (0.8–1.8)	0.02	0.773

Data are expressed as *n* (%) or median (25th and 75th percentiles). *n*: number; r: correlation coefficient; GDF8: growth differentiation factor 8; BMI: body mass index; CAD: coronary artery disease; LVEF: left ventricular ejection fraction; HR: heart rate; SBP: systolic blood pressure; DBP: diastolic blood pressure; STEMI: ST segment elevation myocardial infarction; GRACE: Global Registry of Acute Coronary Events; ICU: Intensive Care Unit; CRP: C-reactive protein; CK: creatine kinase; NT-proBNP: N-terminal pro-brain natriuretic peptide; LDL: low density lipoprotein; HDL: high density lipoprotein; ARB: angiotensin II receptor blockers; ACE inhibitors: angiotensin converting enzyme inhibitors.

**Table 3:** Relevant outcomes according to high/low GDF8 levels (cutoff on median GDF8 value).

	GDF 8 ≤ 2400 ng/L N = 151	GDF 8 > 2400 ng/L N = 145	*p* Value
In-hospital VF/VT	3 (2.0%)	7 (4.8%)	0.211
Troponin Ic peak, ng/mL	8.30 (2.10–36.00)	29.50 (4.22–92.75)	<0.001
LVEF, %	56 (50–60)	55 (50–60)	0.498

**Table 4:** Logistic regression analysis for prediction of troponin I peak.

	Univariate			Multivariate		
	OR	95% CI	*p* Value	OR	95% CI	*p* Value
**Diabetes**	11.82	−3.49–43.03	0.095	12.98	7.86–59.00	0.011
**Smoking**	11.34	0.78–45.40	0.043	13.19	10.32–62.28	0.006
**LVEF > 40%**	19.53	10.88–87.76	0.012	22.23	6.75–94.34	0.024
**GRACE risk score**	0.15	0.16–0.76	0.003	0.168	0.39–1.05	<0.001
**Time to admission**, per min	0.01	−0.02–0.00	0.065	0.01	−0.02–0.01	0.233
**Log GDF8**, per unit	21.59	34.08–119.05	<0.001	26.68	67.05–172.17	<0.001

LVEF: left ventricular ejection fraction; GRACE: Global Registry of Acute Coronary Events; NT-proBNP: N-terminal pro-brain natriuretic peptide; GDF8: growth differentiation factor 8; OR: odds ratio; CI: confidence interval.
